# Supplementary material for: Splice-Junction-Based Mapping of Alternative Isoforms in the Human Proteome
Source: Cell Rep. Author manuscript; Available in PMC 2020 Jan 15. (PMC6961840; doi:10.1016/j.celrep.2019.11.026)
Supplement: 3 [file NIHMS1546469-supplement-3.zip › DF2/PXD009737/TestisLysC-4-Q5T8P6-ERLGPVPSSTIEPAEAQSASSDLPQVLSTSTGLTK.pdf]

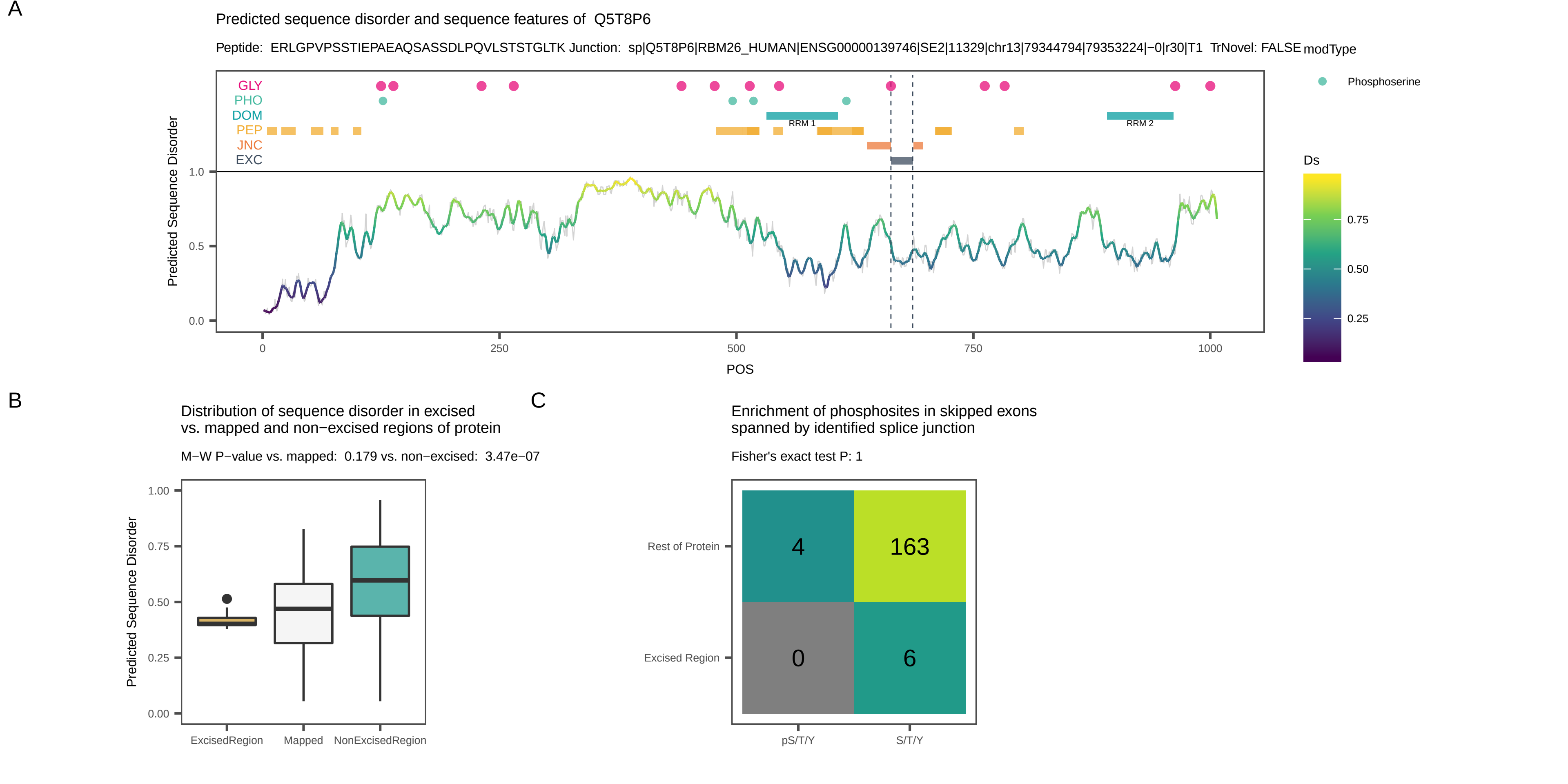
A
Predicted sequence disorder and sequence features of Q5T8P6
Peptide: ERLGPVPSSTIEPAEAQSASSDLPQVLSTSTGLTK Junction: sp|Q5T8P6|RBM26_HUMAN|ENSG00000139746|SE2|11329|chr13|79344794|79353224|−0|r30|T1 TrNovel: FALSE
modType
Phosphoserine
GLY
PHO
DOM
PEP
JNC
EXC
RRM 1
RRM 2
Predicted Sequence Disorder
1.0
0.5
0.0
0
250
500
750
1000
POS
Ds
0.75
0.50
0.25
B
Distribution of sequence disorder in excised
vs. mapped and non−excised regions of protein
M−W P−value vs. mapped: 0.179 vs. non−excised: 3.47e−07
Predicted Sequence Disorder
1.00
0.75
0.50
0.25
0.00
ExcisedRegion
Mapped
NonExcisedRegion
C
Enrichment of phosphosites in skipped exons
spanned by identified splice junction
Fisher's exact test P: 1
Rest of Protein
Excised Region
4
163
0
6
pS/T/Y
S/T/Y
